# Larvae and Nests of Aculeate Hymenoptera (Hymenoptera: Aculeata) Nesting in Reed Galls Induced by *Lipara* spp. (Diptera: Chloropidae) with a Review of Species Recorded. Part II.

**DOI:** 10.1371/journal.pone.0169592

**Published:** 2017-01-11

**Authors:** Alena Astapenková, Petr Heneberg, Petr Bogusch

**Affiliations:** 1 Department of Biology, Faculty of Science, University of Hradec Králové, Rokitanského 62, Hradec Králové, Czech Republic; 2 Third Faculty of Medicine, Charles University, Ruská 87, Prague, Czech Republic; Landcare Research New Zealand, NEW ZEALAND

## Abstract

The ability of aculeate Hymenoptera to utilize wetlands is poorly understood, and descriptions of their nests and developmental stages are largely absent. Here we present results based on our survey of hymenopterans using galls induced by *Lipara* spp. flies on common reed *Phragmites australis* in the years 2015–2016. We studied 20,704 galls, of which 9,446 were longitudinally cut and the brood from them reared in the laboratory, while the remaining 11,258 galls reared in rearing bags also in laboratory conditions. We recorded eight species that were previously not known to nest in reed galls: cuckoo wasps *Chrysis rutilans* and *Trichrysis pumilionis*, solitary wasps *Stenodynerus chevrieranus* and *Stenodynerus clypeopictus*, and bees *Pseudoanthidium tenellum*, *Stelis punctulatissima*, *Hylaeus communis* and *Hylaeus confusus*. Forty five species of Hymenoptera: Aculeata are known to be associated with reed galls, of which 36 make their nests there, and the other are six parasitoids of the family Chrysididae and three cuckoo bees of the genus *Stelis*. Of these species, *Pemphredon fabricii* and in southern Europe also *Heriades rubicola* are very common in reed galls, followed by *Hylaeus pectoralis* and two species of the genus *Trypoxylon*. We also found new host-parasite associations: *Chrysis angustula* in nests of *Pemphredon fabricii*, *Chrysis rutilans* in nests of *Stenodynerus clypeopictus*, *Trichrysis pumilionis* in nests of *Trypoxylon deceptorium*, and *Stelis breviuscula* in nests of *Heriades rubicola*. We provide new descriptions of the nests of seven species nesting in reed galls and morphology of mature larvae of eight species nesting in reed galls and two parasitoids and one nest cleptoparasite. The larvae are usually very similar to those of related species but possess characteristics that make them easy to distinguish from related species. Our results show that common reeds are not only expansive and harmful, but very important for many insect species associated with habitats dominated by this plant species.

## Introduction

Hymenoptera, together with Diptera, Coleoptera and Lepidoptera, represent the four most diverse insect groups, not only according to their species richness but also with regard to variability of life strategies [1–3]. Hymenopterans nesting in various kinds of cavities are related to their various nesting and foraging strategies. Cavity nesters use not only holes in wood, reed stalks or plant stems for their nesting, but they can also adopt quite unexpected places, such as empty snail shells, cavities in old walls or in the reed roofs of buildings. Also, various types of galls host numerous very rare species, represented frequently by narrow habitat specialists [3–6]. Several digger wasps of the family Crabronidae nest in galls induced by the gall wasps of the family Cynipidae [7–9], and a whole group of Aculeata species use the cigar-shaped galls induced by frit flies of the genus *Lipara* (Diptera: Chloropidae) [5–6, 10–12].

The gall-nesting Aculeata are species of various families, which form a specific guild. This ecological group is very heterogeneous, containing reed gall specialists, wetland species (that only occasionally use reed galls for nesting), and ubiquitous species that nest in various types of cavities as well as in reed galls [6]. Although several hymenopterans, which nest in reed galls, are bioindicative, they have been rarely studied. Previous data has often been vague, such as “some species use cigar reed galls for their nesting” [2–4]. Despite being published more than a century ago, many of these works have been used by authors in recent monographic studies on Hymenoptera e.g., [3–4, 13–14]. There have been a variety of species-specific reports, Wolf [10] recorded *Pemphredon fabricii* (that time known as *Pemphredon lethifer*) together with several *Lipara* spp. and their parasitoids in reed galls collected at a single sampling site in Germany. Dely-Draskovits et al. [11] found this species as well as unidentified *Hylaeus* sp. together with many parasitoid species in reed galls collected in Hungary. In southern Germany, six species were recorded by [12]: *Pemphredon fabricii* (as *P*. *lethifer*), *Hylaeus pectoralis*, *Trypoxylon deceptorium* (as *Trypoxylon attenuatum*), *Trichrysis cyanea*, *Hoplitis leucomelana* (as *Osmia leucomelana*), and *Stenodynerus xanthomelas*. In monograph on *Lipara* of Fennoscandia, 26 species of aculeate Hymenoptera and 3 parasitic cuckoo wasps in their nests were reported [15]. Our previous results showed that 13 species nest in reed galls in Czech reed beds located in river floodplains, fishponds and post-industrial sites, and, summarizing all available information, we stated that 29 species are known to nest in reed galls, and were shown to be parasitized by two nest cleptoparasites and four parasitoids of the family Chrysididae [5, 6]. Thus, the community of hymenopterans nesting in reed galls is rich and highly variable.

Recent studies conducted in Europe showed that four species of *Lipara* and their hymenopteran inquilines are distributed across multiple countries [15]. Among their inquilines, *Pemphredon fabricii* is eudominant, usually comprising more than 90% of all aculeate nests and reared aculeate individuals [5–6], followed by *Hylaeus pectoralis* and *Trypoxylon deceptorium*. Most of the other species that have been recorded as nesting in reed galls, are relatively rare and probably use them only occasionally. There are also differences among habitat types, different species occur in large reed beds in fishponds or lake reservations, small sparse reeds on wet meadows, or short-stemmed reed beds in tailing ponds of power stations [see 5].

In this article, we provide a complete list of species recorded in reed galls including sources and countries of occurrence. We focus on aculeate species for which nests and larvae have never been previously described. We also describe, for the first time, the structure of nests and the morphology of mature larvae of eight species that nest in reed galls and three parasitic species. This contribution should thus be considered as a follow-up to our paper published 2015 in this journal [6]. By analyzing an extensive set of reed galls collected across multiple European countries, we were able to collect significant amounts of data even for rare species, which are of special interest for conservation reasons, and thus provide the first available data set on the differences in their larval morphology and nesting preferences.

## Materials and Methods

### Study sites and sampling

**Fig 1:**
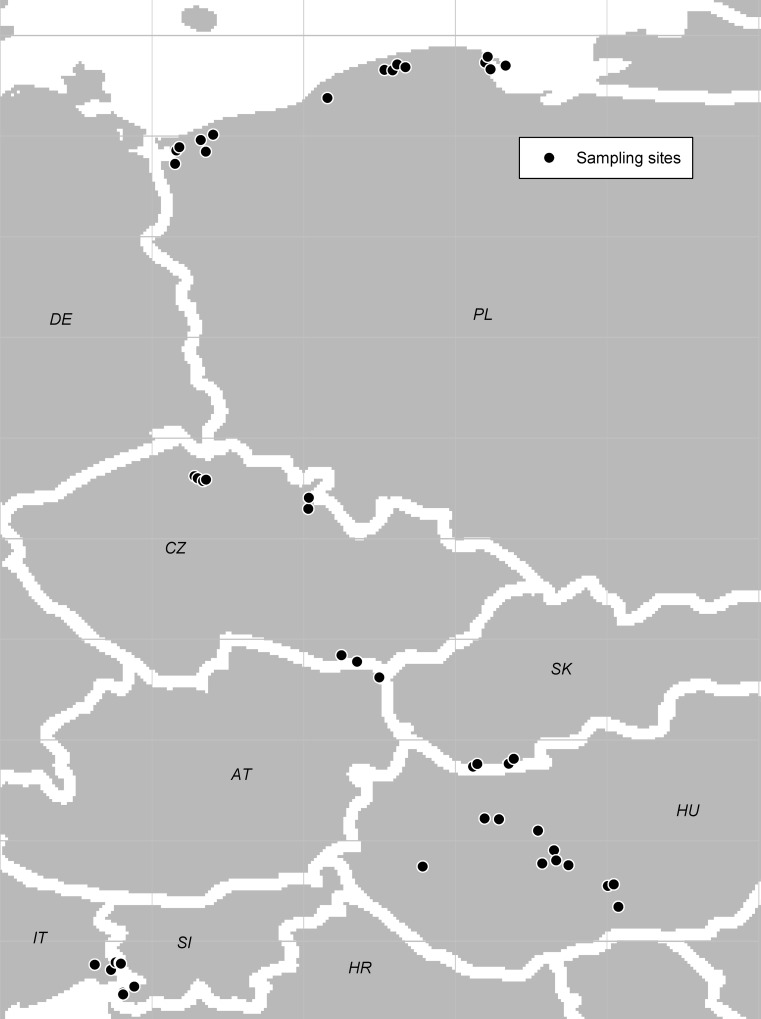
Map of central Europe with the localities studied.

We collected 20,704 galls induced by *Lipara* spp. on common reeds from 47 sampling sites located across central Europe (Fig 1), the sites included: northern Poland (15 sites), Hungary (14 sites), Czech Republic (8 sites), northern Italy (5 sites), Slovenia (3 sites) and Slovakia (2 sites). All localities along with coordinates are listed in S1 Table. Study of plants and animals was possible at all localities without any restriction, except the following: Czolpino, Gardna Wielka, Kluki and Rowy, permission issued by Slowinski National Park headquarters, Smoldzino, Poland, signed by Dr. Ireneusz Izydorek; Apajpuszta, Baks, Izsák, Munkastelep, Orgovány, and Sándorfalva, permission issued by Kiskunság National Park, signed by Ferenc Pál Szabó; Dubno Nature Reserve and Zbytka Nature Reserve, permission issued by Královéhradecký kraj, signed by Jan Novák; Slanisko u Nesytu National Nature Reserve and Slanisko Novosedly Nature Reserve by Pálava Protected Landscape Area, signed by Pavel Dedek; and Břehyně-Pecopala National Nature Reserve, Jestřebské slatiny Nature Reserve, Novozámecký rybník National Nature Reserve and Swamp National Nature Monument, permission issued by Kokořínsko Protected Landscape Area, signed by Věra Lucie Válová. The field studies did not involve any protected animals and no CITES species.

Only galls older than 1 year (greyish or darker in appearance, usually without leaves and often with the apex broken) were collected because our focus was on cavity nesting Hymenoptera (bees and wasps) and not on the *Lipara* spp. (inducing the galls) or their parasitoids. We collected reed galls from 15 Jan to 8 Mar 2015 and additional material from 17 to 22 Jan 2016. In late winter and early spring, mature larvae are present in their nests, and their rearing is easier than if they are collected before hibernation in the autumn [5]. Typically, at least 500 reed galls were collected from each sampling site, of which 200 were longitudinally cut and their contents analyzed, while the remainder were allowed to develop. In the 2016 sampling, only around 200 galls were collected, all of which were cut and none of which were measured. Additionally, galls < 1-year-old (with *Lipara* spp. or their parasitoids present) were removed from analyses, thus the total number of galls analyzed from each site was slightly less than the number collected. At localities with limited availability of galls, we collected only 200–300 galls, which were all either longitudinally cut or reared. To measure dimensions of the cavity within random galls, a subset of less than one year old galls, containing *Lipara* spp. flies, were collected and subsequently measured. In total, 9,446 reed galls were cut and their contents were studied; the other 11,258 were reared in rearing bags in the same way used by [5–6].

### Data acquisition

In the longitudinally cut reed galls, we studied the material of the walls separating the brood cells (henceforth termed bars) and the closing plugs at the top of each nest (henceforth termed corks), the structure and number of brood cells, and also the morphology and coloration of larvae and pupae. In the descriptions, “first cell” means the bottom, i.e., first-built cell of the nest. The “last cell” means the uppermost cell, i.e., the one nearest to the nest entrance. When the larvae were in cocoons, we removed part of the larvae from the cocoons but left the others inside. For each species, we first tried to rear the adults. For nests containing more than three larvae, we conserved part of the brood for morphological studies. To rear the larvale, the living larvae were taken from the nests, placed in Eppendorf 1.5 ml micro-tubes, which were plugged with cotton wool, left at the room temperature with ambient moisture, and reared similarly as described by [6]. The adults usually hatched within three to four weeks after pupation, after which they were fixed, similarly to unreared larvae, i.e., in 96% ethanol. Only members of the family Megachilidae were left to develop in their cocoons, usually for two to three months. We measured the maximum diameter of each reed gall and the diameter of the reed stem just below the reed galls. For a random set of galls with *P*. *fabricii*, and for all well-preserved galls occupied by other aculeate species (except of mixed or parasitized nests), we measured also the length of the reed gall, the length of the nest (from the base of the nest to the cork) and the width of the cavity within the gall.

The reed galls allowed to develop were placed into rearing sacs as described by [5], and allowed to hatch for twelve weeks. The reared individuals were fixed in 20% ethylene glycol solution supplemented with a mixture of ionic and anionic detergents and later transferred to 96% ethanol.

The obtained material was identified by the first author and verified by the last author. Representative specimens (including the nests of each species) are available in the collections of Petr Bogusch (University of Hradec Králové, Czech Republic). We adopted nomenclature used in [14] and [16].

We documented the representative part of the nests using a digital camera (photographs of whole nests) and a macro-photographing apparatus consisting of a macro-camera Canon attached to a stereo microscope (brood cells, whole larvae). Documentation included photographs of nests shared by multiple species of aculeate Hymenoptera and of the parasitized nests. We took photos of living larvae as well as the larvae fixed in Pampel solution (30 volumes of distilled water, 15 volumes of 96% ethanol, 6 of formaldehyde and 4 volumes of glacial acetic acid) as described by [17]. To describe the morphology of larval specimens, we transferred some larvae (but never more than a sample of larvae present in a single nest–part of the larvae were allowed to rear to find out the species) into Pampel solution. After we took the photographs of the intact larvae, we focused on their sclerotized parts. For this purpose, we placed the larvae into 10% solution of hot (60°C) potassium hydroxide, for 12 hours, to dilute all parts of the body except the integument. Then we colored the integument in 5% Chlorazol Black E (Sigma Aldrich) for 2 seconds and moved the specimens into 96% ethanol for conservation. To observe the identification features, we placed the integument into glycerol and separately observed the head, mouthparts, spiracles and other important parts of the integument under a light microscope. We used the same specimens for the study of small structures such as setae, sensillae or mouthparts. We drew figures of (1) the head with a focus on the clypeus, labrum, maxillae, and labium; (2) the mandibles from the anterior view; and (3) the spiracles of larvae of each species.

### Data analysis

The data are shown as means ± SD unless stated otherwise. We analyzed the dataset sampled in 2015 in detail. This included 17,032 *Lipara*-induced reed galls from 34 sampling sites, which were either cut (6,449 galls) or allowed to develop (10,583 galls). Only the species of which we collected five or more individuals were included. We analyzed the occupancy rate of three different size-categories of reed stems and four size-categories of reed galls. To analyze the overall datasets, we employed one-way ANOVA. To analyze the species-specific differences between the observed and expected occupancy rates in the particular size-categories of reed galls and reed stems, we used χ^2^ tests. As “expected frequencies,” we used two groups of comparators, one formed by galls occupied by *Pemphredon fabricii* and the other one formed by random galls collected by our group in 2014. These comparative data sets for gall width and stem width (*Pemphredon fabricii* and random galls data sets) were retrieved from [6].

To analyze the correlations between gall width, stem width, and number of larvae, we calculated linear correlation coefficients r, and Spearman´s D for each species of which we found more than five individuals. To perform this analysis, we merged our data with the data set obtained by [6], which did not perform such analyses but accumulated significant amounts of data. In addition to species-specific correlations, we also calculated the same correlations for random galls (i.e., all those collected in 2014 and 2015). The calculations included the adult specimens of aculeate Hymenoptera as well as their parasitoids obtained in course of this study by hatching the imagines from longitudinally cut galls, and those reared directly from galls. We also calculated Pearson´s correlation coefficients for the inner and outer dimensions of the galls, the numbers of larvae and the ratios of outer to inner dimensions of galls. All calculations were performed using PAST v 2.14; graphs were prepared using SigmaPlot v 8.0.

## Results

### Hymenoptera: Aculeata nesting in cigar galls

Our data, combined with literary sources, suggest that the *Lipara*-induced reed galls host nests of 36 species of Hymenoptera: Aculeata (Table 1). The same nesting resource is also associated with nine species of hymenopteran parasites (six parasitic species of the family Chrysididae and three nest cleptoparasites of the genus *Stelis*).

**Table 1:** Review of all Hymenoptera: Aculeata recorded as nesting in or parasitizing reed galls. Country codes: CZ–Czech Republic, DE–Germany, HU–Hungary, IT–Italy, PL–Poland, SI–Slovenia, SK–Slovakia,??? –unknown. Sources under the numbers used in References chapter.

Family/Species	Country	Literary source
**Chrysididae**		
*Chrysis angustula* Schenck, 1856 *	CZ	5, 6, this study
*Chrysis rutilans* Olivier, 1790 *	HU, SK	this study
*Holopyga fastuosa generosa* Förster, 1853 *	CZ	6
*Pseudomalus auratus* (Linnaeus, 1761) *	CZ	5, 15
*Trichrysis cyanea* (Linnaeus, 1761) *	CZ, DE, HU	5, 6, 12, this study
*Trichrysis pumilionis* Linsenmaier, 1987 *	HU	this study
**Formicidae**		
*Dolichoderus quadripunctatus* (Linnaeus, 1771)	CZ	5
**Vespidae**		
*Stenodynerus chevrieranus* (Saussure, 1855)	HU, IT	this study
*Stenodynerus clypeopictus* (Kostylev, 1940)	HU, SK	this study
*Stenodynerus xanthomelas* (Herrich-Schaeffer, 1839)	DE	12
*Symmorphus bifasciatus* (Linnaeus, 1761)	CZ	5, 6, 15, this study
*Symmorphus fuscipes* (Herrich-Schaeffer, 1839)	???	15
**Crabronidae**		
*Ectemnius confinis* (Walker, 1871)	CZ	5, 6
*Nitela spinolae* Latreille, 1809	CZ	5, 6, this study
*Passaloecus clypealis* Faester, 1947	CZ, PL	5, 6, 15, this study
*Passaloecus corniger* Shuckard, 1837	???	15
*Passaloecus gracilis* (Curtis, 1834)	???	15
*Passaloecus singularis* Dahlbom, 1844	???	15
*Pemphredon fabricii* (Müller, 1911)	CZ, DE, HU, IT, PL, SI, SK	5, 6, 11, 12, this study
*Pemphredon inornata* Say, 1824	???	15
*Pemphredon lethifer* (Shuckard, 1837)	???	12, 15
*Pemphredon rugifer* (Dahlbom, 1844)	???	15
*Pemphredon wesmaeli* (Morawitz, 1864)	???	15
*Rhopalum clavipes* (Linnaeus, 1758)	???	15
*Rhopalum gracile* Wesmael, 1852	CZ, IT	5, 6
*Trypoxylon attenuatum* Smith, 1851	???	12, 15
*Trypoxylon deceptorium* Antropov, 1991	CZ, DE, HU, IT, PL, SK	5, 6, 12, this study
*Trypoxylon figulus* (Linnaeus, 1758)	???	15
*Trypoxylon minus* Beaumont, 1945	CZ, PL, SK	5, 6, this study
**Megachilidae**		
*Chelostoma campanularum* (Kirby, 1802)	CZ	6
*Heriades rubicola* Pérez, 1890	CZ, HU, IT, SI, SK	6, this study
*Hoplitis leucomelana* (Kirby, 1802)	CZ, DE, HU, PL	5, 6, 12, 15, this study
*Megachile centuncularis* (Linnaeus, 1758)	???	15
*Megachile versicolor* Smith, 1844	???	15
*Pseudoanthidium lituratum* (Panzer, 1801)	CZ	6
*Pseudoanthidium tenellum* (Mocsáry, 1881)	HU	this study
*Stelis breviuscula* (Nylander, 1848) *	CZ, SK	6, this study
*Stelis ornatula* (Klug, 1807) *	CZ, PL	6, this study
*Stelis punctulatissima* (Kirby, 1802) *	CZ, HU	this study
**Colletidae**		
*Hylaeus communis* Nylander, 1852	CZ	this study
*Hylaeus confusus* Nylander, 1852	HU	this study
*Hylaeus gracilicornis* (Morawitz, 1867)	???	15
*Hylaeus incongruus* Förster, 1871	CZ	6
*Hylaeus moricei* (Friese, 1898)	CZ	5, 6, 15, this study
*Hylaeus pectoralis* Förster, 1871	CZ, HU, PL, SK	5, 6, 12, 15, 27, this study

* marked are parasitic species.

In this study, we recorded these five species nesting in *Lipara*-induced reed galls for the first time: solitary wasps *Stenodynerus chevrieranus* and *Stenodynerus clypeopictus*, the mason bee *Pseudoanthidium tenellum*, and bees *Hylaeus communis* and *Hylaeus confusus*. Of these species, *S*. *clypeopictus* seems to be especially dependent on nesting in reed galls because this very rare species has been recorded quite frequently in reed galls. We also recorded two new parasitoids: *Chrysis rutilans* in nests of *S*. *clypeopictus* from several localities (Hungary: Bödi-Szék, Orgovány and Sándorfalva and Slovakia: Virt), which is also the first ever record of this host association. We have also recorded *Trichrysis pumilionis* (syn. *Chrysidea pumila*) in nests of *Trypoxylon deceptorium* (Hungary: Sándorfalva, Szabadszállás), and we confirmed previous reports of the nest of cleptoparasite *Stelis punctulatissima* in the nest of *Hoplitis leucomelana* (Hungary: Bödi-Szék), *Chrysis angustula* in the nests of *Pemphredon fabricii* (Czech Republic: Novozámecký rybník), and *Trichrysis cyanea* dominating in the nests of *Trypoxylon deceptorium* and *Trypoxylon minus* (Czech Republic, multiple localities, Slovakia: Virt). We found the parasitoid species, *Trichrysis cyanea* infrequently in the nests of *Pemphredon fabricii* (Czech Republic: Břehyně and Hungary: Orgovány), which also appears to be a novel discovery. We also report for the first time that *Stelis breviuscula* is a nest cleptoparasite of *Heriades rubicola* (Slovakia: Virt) and that *S*. *breviuscula* was very abundant in the nests of *H*. *rubicola* at this (i.e., Slovak) sampling site. We also confirmed the well-known host association of *Stelis ornatula* with *Hoplitis leucomelana*.

The Aculeata associated with *Lipara*-induced reed galls can be divided into three main groups:

1. Species preferring reed galls. These include only *Pemphredon fabricii*, *Hylaeus pectoralis* and probably *Stenodynerus clypeopictus*.
2. Species nesting in reed stalks or other types of cavities and frequently nesting in reed galls. This group is represented by *Trypoxylon deceptorium*, *Hylaeus moricei*, *Heriades rubicola*, and *Passaloecus clypealis*.
3. Species nesting in various types of cavities and accidentally or very rarely nesting also in reed galls. This group includes all other species found so far in reed galls, even though some of these species are quite common in reed galls, this is because they form large populations, of which only a very small percentage use reed galls for nesting. This group is represented by, e.g., *Symmorphus bifasciatus*, *Trypoxylon minus* and *Hoplitis leucomelana*.

The first two groups are sensitive to changes in habitat surrounding reed beds and to the frequency of disturbances affecting the reed beds themselves, therefore they can be used as bioindicators of well-preserved reed beds within intensively cultivated landscapes.

### Structure of nests of selected species

**Fig 2:**
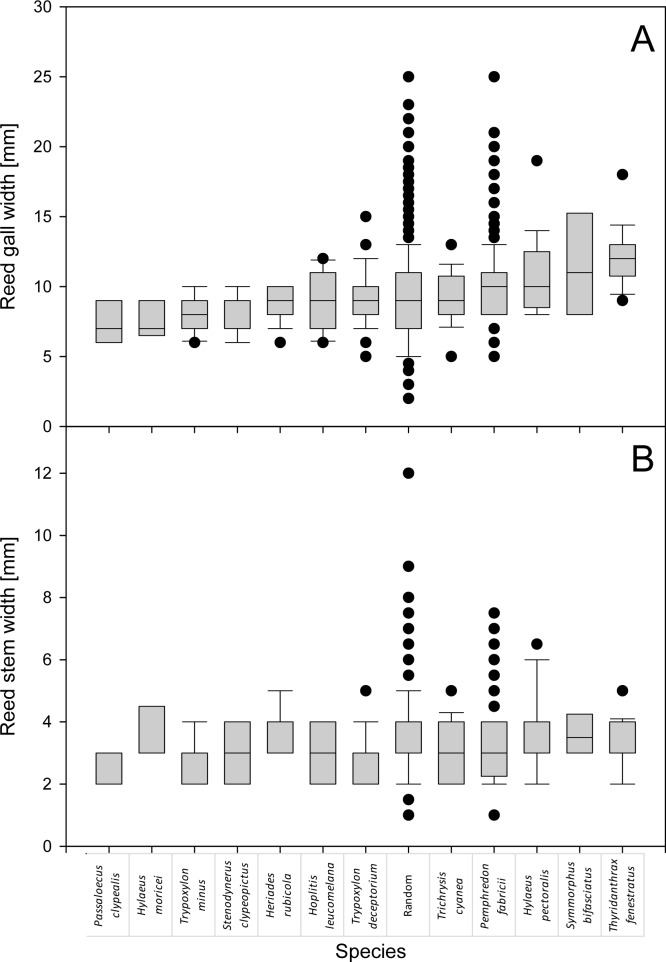
**Species-specific preferences for a particular gall (A) and stem (B) width.** The lines within the boxes show medians, the boxes denote the 25^th^ and 75^th^ percentiles, the whiskers indicate the 10^th^ and 90^th^ percentiles, black points denote outlying points below the 10^th^ and above the 90^th^ percentiles.

In our previous study, we described the structure of nests of the most common species nesting in reed galls [6]. Here, we provide descriptions of nests of seven less common species that also can be found nesting in reed galls, with notes on their differences from nests of similar and related species. The occupancy of nests, by a particular species, changed with the differences in stem width (one-way ANOVA: sum of sqrs = 234.9, d_f_ = 13, F = 11.8, *p* << 0.001), gall width (sum of sqrs = 1031.1, d_f_ = 13, F = 10.0, *p* << 0.001); also the differences in the number of larvae per nest was species-specific (sum of sqrs = 476.2, d_f_ = 12, F = 12.2, *p* << 0.001). We thus analyzed the preferences of particular aculeate hymenopteran species for specific reed gall width, length, and also width of the inner space of the gall and a length of the nest, all correlated with each other, with the number of larvae within the nests and with the gall:nest width and length ratios (Tables 2 and 3, Fig 2A). Besides the data collected in 2015 and 2016 (Table 3), we re-analyzed the data collected in 2014 [6].

**Table 2:** Species-specific preferences for the cavity dimensions. The cavity dimensions were measured as nest length (length of the nest from the base to the plug) and nest width (cavity width), and they were compared with the gall length (Gall:nest length ratio) and width (Gall:nest width ratio). The measures are expressed as means ± SD (range) for N≥3; for lower N, individual measurements are indicated. Note that particularly the *P*. *fabricii* nests extend often out of the cavity and their upper parts may be surrounded by dry leaves only, thus the nests could sometimes be longer than the galls in which they are located. For species with N≥10, the Pearson's correlation coefficients were calculated in order to correlate the nest length and width with the gall length and width, with the number of mature larvae contained within the nests and with the gall:nest length and width ratios.

	Nest length	Nest width	Pearson's correlation coefficient						Gall: nest length ratio	Gall: nest width ratio	Pearson's correlation coefficient				N
			Nest length	Nest width	Nest length	Gall length	Nest width	Gall width			Nest length	Gall length	Nest width	Gall width	
Species			Gall length	Gall width	N of larvae	N of larvae	N of larvae	N of larvae			Length ratio	Length ratio	Width ratio	Width ratio	
*Heriades rubicola*	51.6 ± 13.1 (25–90)	3.9 ± 0.6 (2.5–5)	0.752	0.364	0.657	0.542	0.228	0.063	1.29 ± 0.24 (0.90–1.94)	1.87 ± 0.35 (1.33–3.00)	-0.591	0.061	-0.698	0.398	40
*Hoplitis leucomelana*	34.6 ± 10.4 (20–59)	3.3 ± 0.4 (3–4)	0.783	0.328	0.644	0.533	-0.092	0.304	1.56 ± 0.33 (1.05–2.59)	1.79 ± 0.29 (1.33–2.33)	-0.532	0.077	-0.413	0.721	31
*Hylaeus confusus*	49, 54	3, 3							1.12, 1.24	1.67, 2.00					2
*Hylaeus moricei*	37.7 ± 6.3 (29–44)	3.3 ± 0.5 (3–4)							1.14 ± 0.11 (1.00–1.27)	2.03 ± 0.45 (1.67–2.67)					3
*Hylaeus pectoralis*	44.6 ± 10.8 (23–73)	3.5 ± 0.6 (3–5)	0.726	0.331	0.586	0.464	-0.038	0.322	1.28 ± 0.21 (0.81–1.80)	1.83 ± 0.32 (1.25–2.67)	-0.610	0.073	-0.610	0.537	50
*Passaloecus clypealis*	41.0 ± 2.3 (39–45)	3.8 ± 0.4 (3–4)							1.45 ± 0.19 (1.23–1.73)	1.73 ± 0.04 (1.67–1.75)					4
*Pemphredon fabricii*	50.5 ± 14.4 (12–78)	3.3 ± 0.8 (2–5)	0.271	0.704	0.839	0.175	-0.070	0.054	1.16 ± 0.64 (0.57–5.00)	2.89 ± 0.66 (1.67–6.00)	-0.697	0.210	-0.184	0.555	53
*Stenodynerus clypeopictus*	35.7 ± 13.1 (17–53)	3.6 ± 0.5 (3–4)	0.175	0.684	0.336	-0.356	-0.237	-0.382	1.80 ± 0.94 (1.05–3.76)	1.88 ± 0.27 (1.50–2.43)	-0.839	0.364	0.078	0.777	10
*Symmorphus bifasciatus*	27, 30	3.5, 3.6							1.59, 2.00	1.43, 1.81					2
*Trypoxylon deceptorium*	30.8 ± 8.3 (18–57)	3.2 ± 0.5 (2.5–4.5)	0.432	0.412	0.727	0.210	0.060	-0.092	1.75 ± 0.49 (1.14–3.11)	1.84 ± 0.27 (1.33–2.40)	-0.681	0.302	-0.627	0.433	29
*Trypoxylon minus*	35.3 ± 12.0 (10–70)	3.3 ± 0.6 (2–5)	0.738	0.735	0.394	0.327	0.201	0.223	1.84 ± 0.76 (1.14–5.00)	2.00 ± 0.25 (1.50–2.50)	-0.737	-0.252	-0.540	0.159	29
Random galls with *Lipara* spp.	N/D	3.0 ± 0.71 (2–4.5)		0.438					N/D	2.69 ± 0.69 (1.50–4.50)			-0.545	0.482	50

**Table 3:** Occupancy rate of four reed gall size-categories. The χ^2^ test was employed to compare the observed frequencies (data collected in 2015 during the course of this study) with two types of expected frequencies, namely with the frequencies of galls (i) occupied by *Pemphredon fabricii* and (ii) random galls collected by our group in 2014 [6]. Only species with n ≥ 5 are shown.

Species	Reed gall width				*p* (χ^2^ test)	
	< 5 mm	5–9.5 mm	10–14.5 mm	≥ 15 mm	Obs. vs random	Obs. vs *P*. *fabricii*
*Chrysis angustula*	0	0	1	0		
*Heriades rubicola*	0	14	5	0	3.0 E-01	**4.3 E-02**
*Hylaeus confusus*	0	2	0	0		
*Hylaeus incongruus*	0	0	1	0		
*Hylaeus moricei*	0	5	0	0	2.3 E-01	**5.1 E-02**
*Stelis punctulatissima*	0	1	0	0		
*Stenodynerus clypeopictus*	0	9	2	0	3.0 E-01	**5.3 E-02**
*Trypoxylon minus*	0	26	4	0	**4.0 E-03**	**3.5 E-05**
Comparators:						
*Pemphredon fabricii*	0	469	512	48	**5.4 E-21**	N/A
Random galls	267	3416	2381	287	N/A	**7.0 E-63**

The analysis of gall and nest measurements (Table 2) shows that most of the species use only the inner space of the gall but *Pemphredon fabricii* and in several cases also *Heriades rubicola* and *Hylaeus pectoralis* extend brood cells also to the space between the reed leaves outside of the cavity of the gall. Additionally, *P*. *fabricii* and *H*. *rubicola* have usually longer nests with more brood cells than the others, whereas *Hylaeus pectoralis* also has usually longer nests but with less brood cells because it makes very often empty spaces (false brood cells) inside the gall. *Stenodynerus* spp. and *Trypoxylon* spp. do not use the whole cavity of the gall and they also frequently settle only the top parts of galls, in which the basal parts were settled by another species, usually *P*. *fabricii*. In this regard, it is important to note that we did not find any nest of *S*. *chevrieranus* occupying the whole gall–in both two nests examined, this species settled in the empty space of the gall pre-occupied in part by *P*. *fabricii*). The ratio of gall versus nest lengths did not differ significantly among most of the species, with the exception of *Trypoxylon minus*, which usually made short nests in long galls. Also the ratio of gall versus nest widths did not show any species-specific pattern except of *P*. *fabricii* and *S*. *clypeopictus*, which preferred equally wide cavities in thin as well as in wide galls. All other species settled in galls, which displayed positive correlation of the cavity width with the gall:nest width ratio. Thus, only *P*. *fabricii* and *S*. *clypeopictus* settled narrow galls as long as the cavity proportions were useful for them, which was not recorded in the other species.

**Fig 3:**
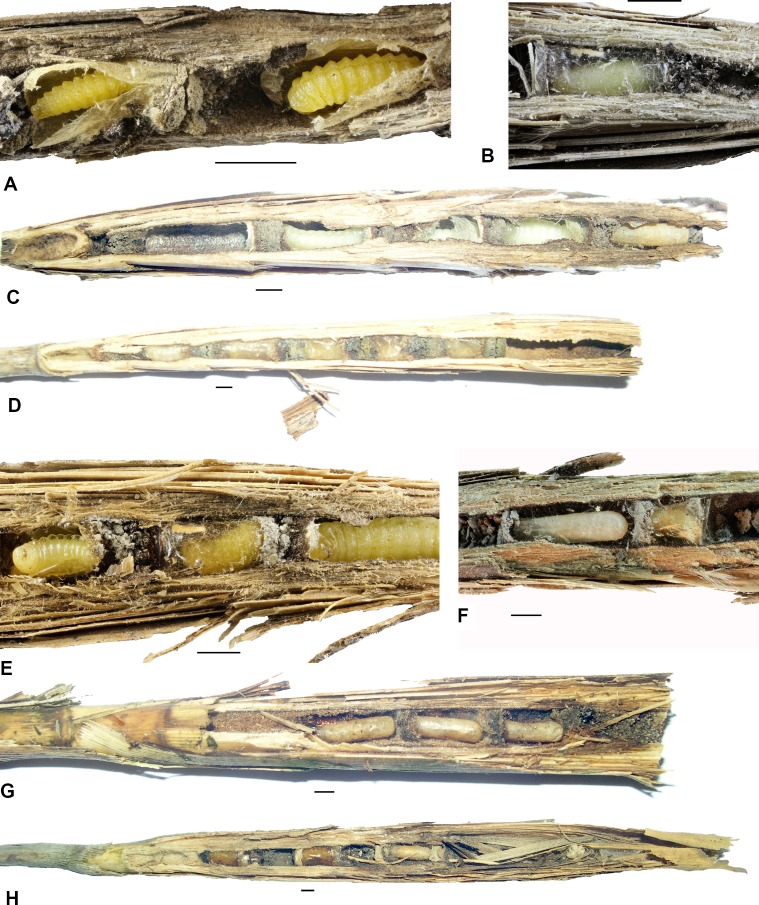
Photos of nests and parts of the nests of aculeate Hymenoptera in reed galls. A–*Symmorphus bifasciatus*, part of a nest with two larvae in cocoons, B–*Stenodynerus chevrieranus*, brood cell, C–*Stenodynerus clypeopictus*, nest with three white colored larvae and *Trypoxylon* sp. in the last brood cell, D–*S*. *clypeopictus*, nest with five brood cells, E–*S*. *clypeopictus*, details of a nest with one larva of this species and two larvae of *Chrysis rutilans*, F–*Trypoxylon deceptorium*, details of a nest with one larva in a cocoon and one short cocoon with larva of *Trichrysis pumilionis*, G–*T*. *minus*, nest with three brood cells, H–*T*. *minus*, nest with one cocoon of this species and two cocoons of *Trichrysis cyanea*. Measurements show 2 mm.

**Fig 4:**
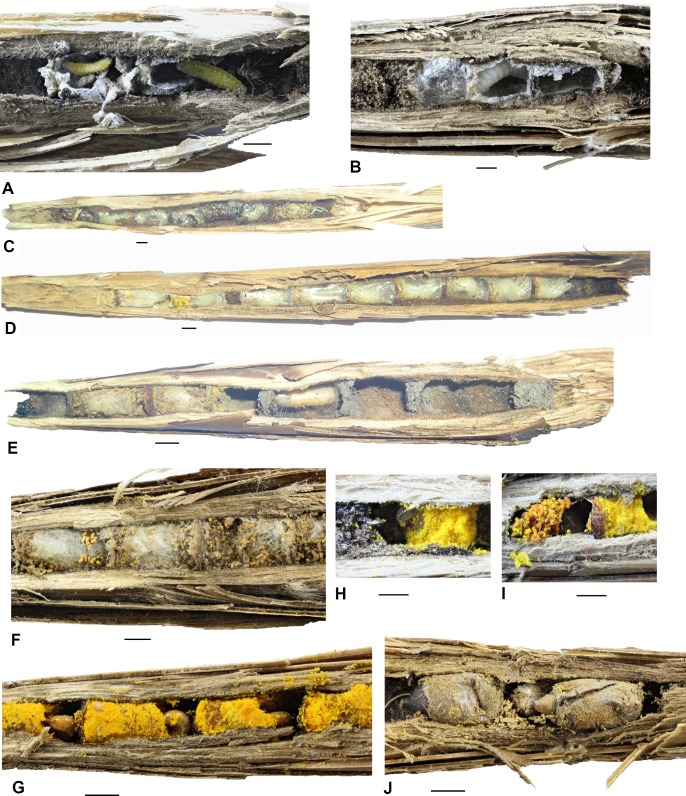
Photos of nests and parts of the nests of aculeate Hymenoptera in reed galls. A–*Passaloecus clypealis*, nest with two brood cells with yellow larvae, B–*Hylaeus moricei*, details of a nest with three brood cells with one larva, C–*H*. *moricei*, nest with six brood cells, D–*Heriades rubicola*, nest with nine brood cells, E–*H*. *rubicola*, nest with two brood cells and the rest full of brood cells and one cocoon of *Trypoxylon* sp., F–*H*. *rubicola*, details of a nest with three brood cells and larval feces on their surface, G–*H*. *rubicola*, details of brood cells with young larvae on yellow pollen, H–*H*. *rubicola*, brood cell with young larva, I–*H*. *rubicola*, brood cell with premature larva with the rest of the pollen and feces, J–*Stelis breviuscula*, brood cells of characteristic shape in a nest of *H*. *rubicola*. Scale bars show 2 mm.

The analysis of width of galls and stems indicated that some species are associated with galls of specific width. Particularly, *Symmorphus bifasciatus* and *Thyridanthrax fenestratus* were associated characteristically with galls of significantly smaller diameter compared to both random galls or galls occupied by *Pemphredon fabricii*. We also analyzed the preferences of particular aculeate hymenopteran species for specific reed stem width (Table 4, Fig 2B). Besides the data collected in 2015 (Table 4), we re-analyzed the data collected in 2014 [6], which indicated that some aculeate species differ in their stem width preferences. In particular, *Hylaeus pectoralis* was associated with the widest available stems, and thus differed significantly from *Pemphredon fabricii*. *Trypoxylon deceptorium* was more prevalent in thin stems, and thus differed significantly in this parameter from randomly collected reed galls; this was also true for *Trypoxylon minus* and *Pemphredon fabricii*. In multiple species, the number of larvae per nests correlated with the reed gall width but not with the reed stem width (Table 5), which is consistent with the preferences of most species for galls induced by *Lipara lucens*, which forms thick galls even on very thin stems. Such correlations suggest that females of these species are limited by the dimensions of available cavities, i.e., galls. Photos of nests of all species are in Figs 3 and 4.

**Table 4:** Occupancy rate of three reed stem size-categories. The χ^2^ test was employed to compare the observed frequencies (data collected in 2015 and 2016 during the course of this study) with two types of expected frequencies, namely with the frequencies of galls i) occupied by *Pemphredon fabricii* and ii) random galls collected by our group in 2014 [6]. Only species with n ≥ 5 are shown.

Species	Reed stem width			*p* (χ^2^ test)	
	> 4 mm	4–5.5 mm	≥ 6 mm	Obs. vs random	Obs. vs *P*. *fabricii*
*Chrysis angustula*	1	1	0		
*Heriades rubicola*	16	13	0	1.6 E-01	**2.3 E-02**
*Hylaeus confusus*	2	0	0		
*Hylaeus incongruus*	1	0	0		
*Hylaeus moricei*	4	2	0	8.1 E-01	8.2 E-01
*Stelis punctulatissima*	1	1	0		
*Stenodynerus clypeopictus*	11	3	0	3.7 E-01	8.8 E-01
*Trypoxylon minus*	30	5	0	**1.2 E-02**	3.2 E-01
Comparators:					
*Pemphredon fabricii*	953	298	18	**3.9 E-25**	N/A
Random galls	4899	2515	506	N/A	**0.0 E+00**

**Table 5:** Correlation analysis of the number of larvae per nest with gall dimensions suggests that gall size is the limiting factor for females of aculeate hymenopterans. To analyze the correlations of gall width, stem width, and number of larvae, we calculated linear correlation coefficients r, and Spearman´s D for each species with more than five individuals. To perform this analysis, we merged our data with the data set obtained by [6]. Besides the species-specific correlations, we also calculated the correlation for random galls (all those collected in 2014 and 2015).

	Linear correlation r						Spearman´s D					
	Gall width		Gall width		No. of larvae		Gall width		Gall width		No. of larvae	
Species	vs. No. of larvae		vs. stem width		vs. stem width		vs. No. of larvae		vs. stem width		vs. stem width	
*Heriades rubicola*	−0.05		0.59	**	0.01		1109		399	*	971	
*Hoplitis leucomelana*	0.72	*	0.57		0.28		40	*	69		109	
*Hylaeus pectoralis*	0.38		0.61	***	0.07		2043		1268	**	2775	
*Pemphredon fabricii*	0.45	***	0.56	***	0.32	***	1.0E+08	***	7.2E+07	***	1.2E+08	***
*Stenodynerus clypeopictus*	−0.36		0.64	*	0.00		245		68	*	188	
*Symmorphus bifasciatus*	0.92	**	0.74		0.50		6		8		8	
*Trichrysis cyanea*	0.52	*	0.36		0.14		296	*	362		488	
*Trypoxylon deceptorium*	0.59	***	0.49	**	0.09		4305	***	4450	**	8050	
*Trypoxylon minus*	−0.02		0.45		0.02		37		17		33	
Random galls	N/A		0.44	***	N/A		N/A		2.5E+10	***	N/A	

Asterisks show the significance of the results–

* significant

** highly significant

*** very highly significant.

#### Stenodynerus clypeopictus

The nests of *Stenodynerus clypeopictus* usually consisted of 1–2 brood cells (range 1–4; median 1; mean 1.8 ± 1.07 cell per nest; n = 10). The brood cells were quite long (length 8.1 ± 0.91 mm; median 8 mm; width 3.3 ± 0.42 mm; median 3.25 mm; n = 19) and were separated by bars made of soil, sometimes mixed with plant debris (approximately 3 mm thick). In some cases, the soil bar was surrounded on both sides by a layer of plant debris mixed with larval feces. The nests were made at the base of the gall cavity. However, in galls with a very narrow base, the brood cells were placed in the wider part of the gall, and the base of the gall was not filled with anything. The brood cells were placed in the gall one after the other. When there were smaller numbers of cells, the upper part of the gall was filled with a substrate consisting of soil and small sand or silt grains (smaller than 0.5 mm). The surface of the brood cells was covered by a shiny silk-like layer made by secretions from the female’s Dufour’s glands, the layer on the inner surface of the brood cell was creamy-white, bright and opaque, and made from the silk of mature larvae (Fig 3B–3E).

We recorded cuckoo wasps, *Chrysis rutilans*, in the nests of *S*. *clypeopictus* at three localities in Hungary (Bödi-Szék, Orgovány, Sándorfalva) and one locality in the south of Slovakia (Virt). This species was discovered to be a parasite of *S*. *clypeopictus* and seems to typically be a parasite of small-sized species of solitary wasps. *Chrysis rutilans* had shiny and slightly conical cocoons, which were glued to the bars on the bottom side but not glued on the upper side. The cocoons were transparent and significantly shorter than brood cells of *S*. *clypeopictus*. Both ends of the cocoons were reddish brown (Fig 3E).

#### Stenodynerus chevrieranus

We found only one nest of *Stenodynerus chevrieranus*. It consisted of 2 brood cells and was located near Fonyód, close to Lake Balaton in Hungary. The nest was at the end of a gall occupied by *Pemphredon fabricii*. Brood cells were covered by a shiny creamy-white layer, very similar to that of *S*. *clypeopictus*. The bars between the brood cells were made of soil and were only around 1 mm thick (shorter than at *S*. *clypeopictus*). The end of the nest was filled with soil and grit.

#### Symmorphus bifasciatus

Two nests of this species, from the 2016 season, were comprised of two and three brood cells. They were very similar to those previously described by [6]. The bars between the brood cells were made of soil and small grit. The quite thick cork (3–4 mm) was made of soil and there were no empty cells in the gall. Light-brownish cocoons were made of silk, and slightly extended toward the head of the pupa. The cocoons did not fill the whole brood cell, but rather only about two thirds of it. We found remnants of larvae and adults of chrysomelid beetles (*Galleruca* sp.) in the brood cells (Fig 3A).

#### Passaloecus clypealis

*Passaloecus clypealis* nests were very similar in their general appearance with those of *Pemphredon fabricii* but had much smaller brood cells (length 5.5 mm ± 0.55 mm; median 5.2 mm; width 2.8 mm ± 0.54 mm; median 2.8 mm; n = 7). The nests were comprised of 4–6 brood cells (median 4; mean 4.5 ± 0.87 cell per nest; n = 4 nests). Between the brood cells, there was usually a filling of large (0.5–0.8 mm) grit glued by soil and other unidentified materials. The walls of the brood cells were covered with a white silk-like layer that created white cocoons made by the larva after it defecated. The base of the nest was filled with grit; the cork was made of grit, silk, and aphid parts. Brood cells were separated from each other by ≈1 mm thick bars of soil. The empty parts between the brood cells were filled with unidentified materials, probably a mixture of larval feces and soil particles. Larvae pupated in the brood cells covered with silk but without making cocoons (Fig 4A).

#### *Trypoxylon deceptorium* and *T*. *minus*

Nests of both species were described by [6]. We also improved our knowledge of *T*. *minus* nests based on additional material from Poland and the Czech Republic. We usually recorded 1–3 brood cell per nest (range 1–4; median 2; mean 2.2 ± 0.99 cells per nest; n = 28). The nest structure was similar to that described by [6]. We also recorded the cuckoo wasp *Trichrysis cyanea* as a parasitoid in nests of *T*. *minus* (see Figs 2F–2H and 4E).

We confirmed the cuckoo wasp *Trichrysis pumilionis* as a parasite in the nests of *T*. *deceptorium* in Hungary (Sándorfalva and Szabadszállás). The cocoons of this cuckoo wasp had the same structure as cocoons of *T*. *cyanea*, which parasitizes nests of *Trypoxylon* spp. across central Europe. However, only one larva of *T*. *pumilionis* was in each of the nests, so we did not have sufficient data to describe the morphology of the larva of this species.

#### Heriades rubicola

The nests of *Heriades rubicola* usually consisted of 3–4 brood cells, less frequently up to 7 (range 1–7; median 4; mean 3.7 ± 1.6 cell per nest; n = 23). Brood cells (length 6.1 mm ± 0.88 mm; median 6 mm; width 3.6 mm ± 0.48 mm; median 3.5 mm; n = 61) were placed in the cavity one after another and separated from each other by very thin bars (less than 1 mm) consisting of the same material as that filling the gall. The nests comprised the whole gall with no empty cells as protection against parasitoids. The top of the gall was filled with a substance made of resin and chewed plant tissues (light brown pieces of reed or grass leaves). The cork was just behind the last cell and was made of silt grains and resin (hard and sticky mass). In some cases, the narrow base of the gall was filled with silt grains. Mature larvae were placed in brownish, silk and cellophane-like cocoons (similar to cocoons of *Hoplitis leucomelana*). The cocoons were hard (not easy to open) and made of silk. Imagines hatched about 2–3 months after pupation, which is a very long time in comparison to other species. The males hatched first, about 1–2 weeks before females. Nests of this species consisted of the horn-shaped feces of the larvae, which remained on the surface of the cocoons and were often found to be getting moldy (Fig 4D–4F). Nests with young larvae collected in summer were full of brood cells with yellow-orange pollen of Asteraceae. The brood cells were filled to one-half to two-thirds of their volume with pollen. Larvae fed directly on the pollen, higher instars had a brownish coloration (Fig 4G–4I).

At Virt in southern Slovakia, nests of *H*. *rubicola* were frequently parasitized by *Stelis breviuscula* (seven of 45 nests of *H*. *rubicola* were parasitized by 15 individuals of *S*. *breviuscula*), whose brood was typically placed in brownish oval cocoons with a cusp on the bottom and on the top (Fig 4J).

#### Hylaeus confusus

The nests of *Hylaeus confusus* were very similar to *H*. *pectoralis;* we did not find any significant differences. Two nests found in our survey were comprised of two and three brood cells.

#### Hylaeus moricei

The nests of *Hylaeus moricei* were very similar to the nests of *H*. *pectoralis* but smaller and usually with more brood cells (two nests of our dataset comprised three and six brood cells), whose length was 5.0 mm ± 1.24 mm; median 4.3 mm; and width 3.2 mm ± 0.39 mm; median 3.2 mm; n = 9. The nests usually did not occupy the whole gall but no empty cells were placed between the brood cells with larvae. The bases of the galls were filled with chewed plant particles (> 1 mm). Above the filling was a space of very similar size to that of brood cells, then chewed plant particles again, and then brood cells that were only separated by thin cellophane-like layers. The walls of the brood cells were also covered by a cellophane-like material, which was made by the female and consisted of secretions from her Dufour’s gland. At the end of the gall from Novozámecký rybník, there was a cork made of plant particles and mud and elongated pieces of leaves (possibly reed leaves) on top. Mature larvae were very close to each other, but without cocoons (Fig 4B and 4C).

### Description of mature larvae

**Fig 5:**
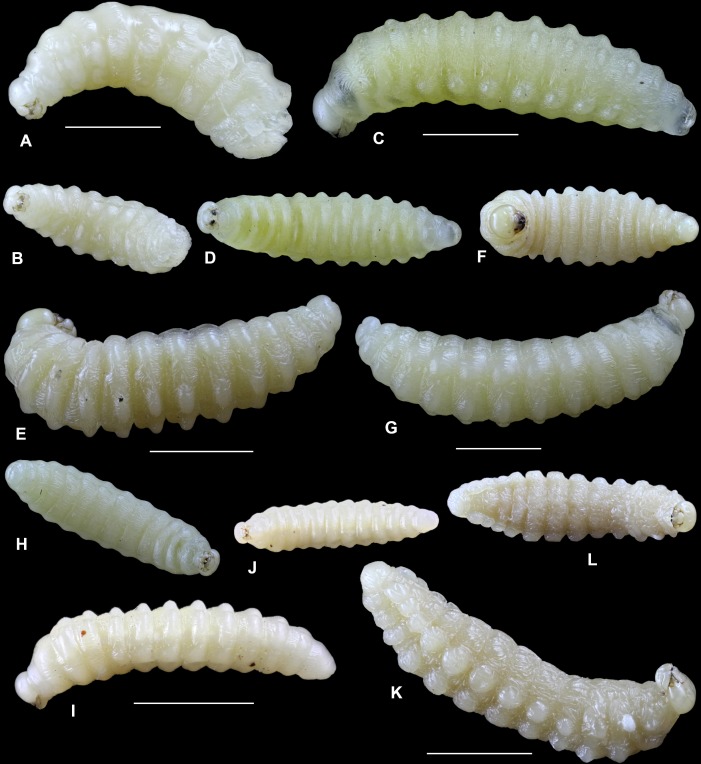
Larvae of aculeate Hymenoptera in reed galls. A–B–*Chrysis rutilans*, lateral and ventral view; C–D–*Symmorphus bifasciatus*; E–F–*Stenodynerus chevrieranus*; G–H–*S*. *clypeopictus*; I–J–*Passaloecus clypealis*; K–L–*Trypoxylon minus*. Measurements show 2 mm.

**Fig 6:**
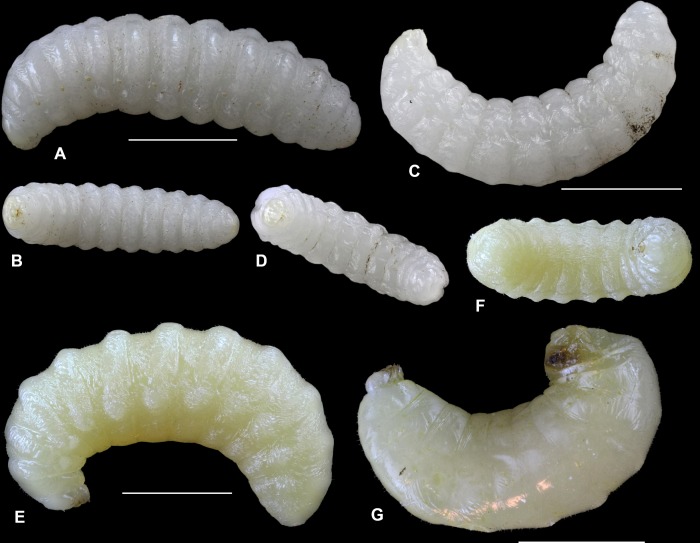
Larvae of aculeate Hymenoptera in reed galls. A–B–*Hylaeus confusus*, lateral and ventral view; C–D–*Hylaeus moricei*; E–F–*Heriades rubicola*; G–*Stelis breviuscula*. Measurements show 2 mm.

**Fig 7:**
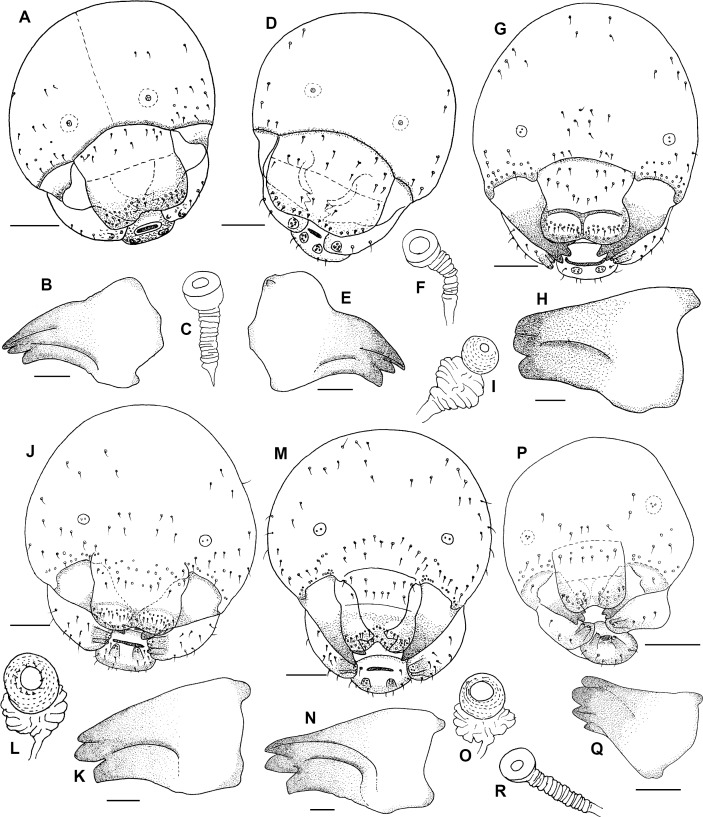
Morphology of larvae of aculeate Hymenoptera in reed galls. A–C–*Chrysis angustula*; D–F–*Chrysis rutilans*; G–I–*Symmorphus bifasciatus*; J–L–*Stenodynerus clypeopictus*; M–O–*Stenodynerus chevrieranus*; P–R–*Passaloecus clypealis*, all species head capsule frontal view, mandible lateral view, spiracle. Measurements show 0,2 mm in drawings of heads and 0,1 mm in drawings of mandibles.

**Fig 8:**
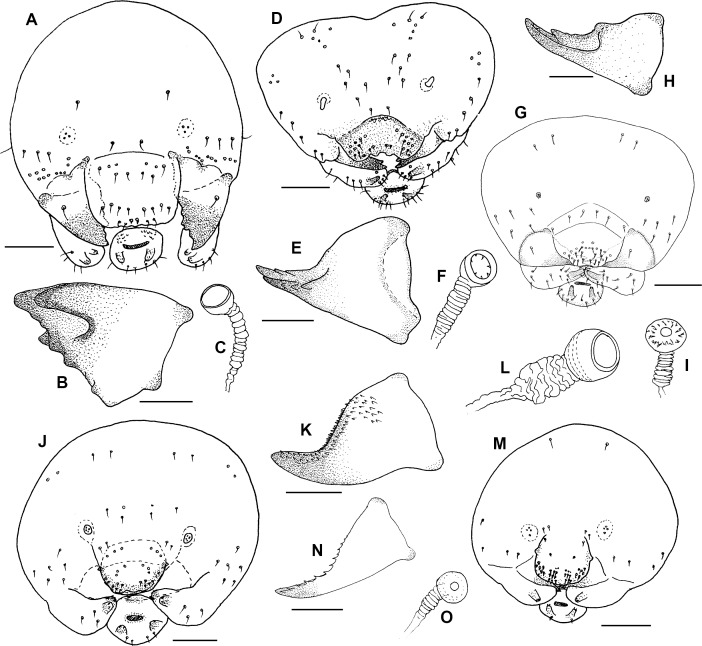
Morphology of larvae of aculeate Hymenoptera in reed galls. A–C–*Trypoxylon minus*; D–F–*Heriades rubicola*; G–I–*Stelis breviuscula*; J–L–*Hylaeus confusus*; M–O–*Hylaeus moricei*, all species head capsule frontal view, mandible lateral view, spiracle. Measurements show 0,2 mm in drawings of heads and 0,1 mm in drawings of mandibles.

We analyzed mature larvae of eight species of aculeate Hymenoptera nesting in reed galls induced by *Lipara* spp., two of their parasitoid species, and one nest cleptoparasite found in their nests. Below, we provide descriptions of mature larvae, including photos of whole larvae from lateral and ventral views (Figs 5 and 6) and drawings of the main identification characteristics–head capsules, mandibles, and spiracles (Figs 7 and 8).

#### *Chrysis angustula* (Fig 7A–7C)

Mature larvae of this species have been described previously by [18]. However, the species was divided into two species later by [19] so we cannot state larva of which of them was described previously.

Material: Czech Republic, Bohemia bor., Zahrádky, Novozámecký rybník National Natural Reserve, terrestrial reed bed surrounding fishpond, 08.iii.2015, 1 larva, P. Bogusch et A. Astapenková lgt., P. Bogusch det. (coll. P. Bogusch).

Body: Short and robust, white colored, length 5.92 mm; width 2.22 mm (n = 1). Fusiform in shape with well-developed dorsal posterior lobes reaching pleurae of segments. Pleural lobes well developed and forming a line. Not dorsoventrally flattened. Last abdominal segmentrounded, very short, and narrower than other segments. Anus terminal is a transverse slit. Integument almost smooth, bearing only a few short setae. Spiracles well sclerotized, brownish; atrium has a very wide margin (more than half the width of the pore), short, with only one septum.

Head and mouthparts: Head well visible, more than half the width of the abdominal segments. Rounded, with typical frontoclypeal suture in the middle, width 0.91 mm, height 1.15 mm, width:height ratio < 1. Pale and unpigmented except for brownish marking on the following structures: antennal orbits, frontoclypeal suture, anterior and posterior tentorial arms, labrum, teeth and joints of mandibles, some parts of the maxillae, and labium. Antennal orbits clearly visible, with four very small sensory cones in the membrane. Head with punctures bearing setae, mostly on vertex, above antennal orbits and above joints of mandibles. Clypeus and labrum not visibly separated, clypeus less sclerotized, on apical part of clypeus 12 setae (six on each side). Labrum longer than clypeus, sclerotized especially on the apical part, with slight emargination in the middle, with 16 sensillae on each side, five of them bearing setae. Sensillae located near apical lobes of labrum but mostly on the sides. Mandible (length 0.41 mm) with at least three teeth; apical tooth is longer and sharper than the other teeth. Maxillae with four conspicuous setae on each side. Galea short but easily visible, sclerotized, with five sensillae, one of them elongated. Maxillar palpus short and smaller than the galea, with two short sensillae at the distal end. Labium rugous has a wide salivary slit. Labial palpus sclerotized and short, with four conical sensillae and one elongated sensilla.

#### *Chrysis rutilans* (Figs 5A, 5B and 7D–7F)

Mature larvae of this species have not been previously described.

Material: Hungary centr., Kiskunság National Park, Orgovány env., terrestrial reed bed, 24.ii.2015, 2 larvae; Dunatetétlen env., Bödi-Szék, salt marsh and terrestrial reed bed, 24.ii.2015, 2 larvae; Hungary mer., Kiskunság National Park, Sándorfalva env., terrestrial reed bed, 25.ii.2015, 1 larva, all P. Bogusch et P. Heneberg lgt., all P. Bogusch det. (coll. P. Bogusch).

Body: Short and robust, widest in the hind part, white colored, length 5.72 ± 0.59 mm; width 2.12 ± 0.04 mm (n = 5). Fusiform in shape with well-developed dorsal posterior lobes reaching pleurae of segments. Pleural lobes well developed and forming a line. Not dorsoventrally flattened. Last abdominal segment very short, rounded, and narrower than other segments. Anus terminal is a transverse slit. Integument almost smooth, with only a few short setae. Spiracles sclerotized, brownish, with a wide margin (margin wider than half the width of the atrium). Atrium with one septum, shorter than wide.

Head and mouthparts: Head large but slightly narrower than thorax, longer than wide: width 1.00 mm, height 1.17 mm, width:height ratio < 1. Frontoclypeal suture in the middle. Most of the head pale and unpigmented except for brownish markings on the following structures: frontoclypeal suture, clypeus (only very slightly), labrum, teeth and joints of the mandibles, apex of the maxillae and labium. Antennal orbits small, unsclerotized, with three sensory cones in the membrane. Six setae on each side of the head, most of them above the joints of the mandibles. Clypeus two and a half times longer than it is wide, with two setae on the apex and four setae in the middle on each side. Labrum shaped like a rounded rectangle, slightly sclerotized, with only slight serrations on the apical margin, without setae and sensillae. Five prominent setae on each side of labrum and a row of small sensillae posterior to them. Mandible (length 0.38 mm) has three teeth, with the ones on the outer side being a bit longer and sharper. Maxillae blunt, poorly sclerotized at the ends, with three conspicuous setae on each side. Galea short but easily visible, with four short sensillae and one elongated sensilla. Maxillar palpus poorly visible with one large and one small sensilla. Labium contained a wide salivary slit, with rough structures at the end and three setae on each side. Labial palpi have four conical sensillae and one elongated sensilla. Hypopharynx easily visible, not serrated.

#### *Stenodynerus chevrieranus* (Figs 5E, 5F and 7M–7O)

Mature larvae of this species have not been previously described.

Material: Hungary occ., Fonyód env., reed bed near Lake Balaton, 25.ii.2015, 2 larvae, P. Bogusch et P. Heneberg lgt., P. Bogusch det. (coll. P. Bogusch).

Body: Length 7.7–7.8 mm; width 2.0–2.1 mm (n = 2). Yellow or yellowish in color. Posterior parts of segments have distinct lobes, pleural lobes also well developed, pleural lobes less distinct on the prothorax and mesothorax and most distinct on the central abdominal segments. Last abdominal segment larger and knob-shaped, with a transverse slit anus. Integument forms a fine cuticle with smooth parts, every segment bears tens of small sensillae. Spiracles well developed, funnel shaped, with a very narrow margin around the atrium, in six unbroken lines.

Head and mouthparts: Head rounded with a suture in the middle of the vertex, suture failed to reach the clypeus. Head longer than wide, width 1.09–1.1 mm, height 1.22–1.25 mm, width:height ratio < 1. Head pale and unpigmented except for brownish markings on the following structures: antennal orbits, apical part of clypeus, labrum, mandibles, maxillae, galeae, and labium. Antennal orbits large and rounded with three sensillae. Head has punctures bearing setae, mostly on the frons and above mandibular joints. Three groups of small sensillae (3 + 3 + 7) are present, but only on the mandibular joints. Clypeus wider than long, apical part sclerotized and rugous with six sensillae bearing setae on each side. Labrum sclerotized, its margin has two lobes on each side, a depression in the middle with numerous sensillae. Labrum has more than 15 sensillae bearing setae on each side. Epipharynx with small teeth. Mandible (length 0.49 mm) with three large teeth of similar size, inner tooth shorter, blunt, and quadratic. Maxillae slightly sclerotized with nine interspersed conspicuous setae. Galea elongated, well sclerotized, brownish, and with three conical sensillae and one elongated sensilla in the apical part. Maxillar palpus wider than galea, narrowing sharply toward the apex, with two small sensillae at the apex. Labium sclerotized and curved, wide salivary slit, and five setae on each side. Labial palpus short, cylindrical, with three conical sensillae and one elongated sensilla.

#### *Stenodynerus clypeopictus* (Figs 5G, 5H, 7J and 7K)

Mature larvae of this species have not been previously described.

Material: Hungary centr., Kiskunság National Park, Orgovány env., terrestrial reed bed, 24.ii.2015, 7 larvae; Dunatetétlen env., Bödi-Szék, salt marsh and terrestrial reed bed, 24.ii.2015, 7 larvae; Hungary mer., Kiskunság National Park, Sándorfalva env., terrestrial reed bed, 25.ii.2015, 1 larva, all P. Bogusch et P. Heneberg lgt., all P. Bogusch det. (coll. P. Bogusch).

Body: Length 9.57 ± 1.27 mm; width 2.22 ± 0.19 mm (n = 15). Color yellow or yellowish but in some cases also white or whitish. Elongated, slim, dorsoventrally flattened body. Posterior parts of the segments on the dorsal part form distinct lobes, pleural lobes also well developed, pleural lobes less developed on the prothorax and mesothorax and largest on the central abdominal segments. The last abdominal segment rounded with a transverse slit anus. Integument smooth with small inconspicuous setae.

Head and mouthparts: Head smallish, only slightly wider than half the width of the thoracic segments, width 1.22 mm, height 1.38 mm, width:height ratio < 1. Head has a suture on the vertex, which failed to reach the clypeal margin. Head pale and unpigmented except for brownish markings on the following structures: antennal orbits, clypeus and labrum (only very slightly), teeth and joints of mandibles, maxillae, galeae, maxillar palpi, and labium. Antennal orbits flat with three sensory cones in the membrane. Head has many punctures bearing setae, mostly on the vertex, around antennal orbits, and near joints of the mandibles. Clypeus short, length:width ratio 1:4, the front part straight, not sclerotized, bearing five setae and nine sensillae on each side. Labrum with two lobes slightly raised, depression in the middle, 12 setae and at least 18 sensillae on each side. Epipharynx has numerous tooth-like processes. Mandible (length 0.50 mm) with three conspicuous rounded teeth. Maxilla rounded, slightly sclerotized, with nine setae on each side. Galea conspicuous and sclerotized, with three conical sensillae and one elongated sensilla. Maxillar palpus narrowed towards the end, longer than galea, with two sensillae at the end. Labium with wide salivary slit (width almost equal to the whole labium), with ten setae on each side. Labial palpus bearing threelarge and one smaller sensillae.

Remarks: Spiracles very similar to those of *S*. *chevrieranus*.

#### *Symmorphus bifasciatus* (Figs 5C, 5D and 7G–7I)

Mature larvae of this species have not been previously described.

Material: Czech Republic, Bohemia centr., Mělník env., terrestrial reed bed surrounding complex of abandoned sandpits, 22.ix.2015, 3 larvae, P. Bogusch et A. Astapenková lgt.; Bohemia or., Rzy, terrestrial reed bed surrounding fishpond, 31.xii.2015, 2 larvae, P. Bogusch lgt., all P. Bogusch det. (coll. P. Bogusch).

Body: Length 7.9–8.1 mm; width 2 mm (n = 2). Yellowish in color. Posterior parts of the dorsal segments form distinct lobes, pleural lobes also well developed, pleural lobes less developed on the prothorax and mesothorax and largest on the central abdominal segments. All lobes much less distinct than in both *Stenodynerus* species. The last abdominal segment knob-like with a transverse slit anus. Integument rough, with many very small spinules (well visible under 400× magnification). Spiracles easily visible, brownish, with a round atrium. Atrial margin very thin, with seven lines.

Head and mouthparts: Head easily visible, narrower than thorax, width 1.10 mm, height 1.32 mm, width:height ratio < 1. Head with suture on the vertex towards the frons, but not reaching the clypeal margin. Head pale and unpigmented except for brownish markings on the following structures: antennal orbits, clypeus, labrum, teeth and joints of mandibles, maxillae, and labium. Large antennal orbits, with two sensillae. Head with many pits bearing setae, mostly on the vertex, above the clypeus and joints of the mandibles. Clypeus long, sclerotized, but only at the base and at the end; the basal clypeal suture well developed. Eight setae on each side of the clypeus. Labrum shorter than clypeus, with a depression in the middle full of setae and sensillae. Apical margin of labrum and central part is sclerotized. Labrum with two main lines of setae and sensillae, several sensillae also located near the apical margin (altogether ten setae and 15 sensillae). Epipharynx with tooth-like processes. Mandible (length 0.49 mm) straight, with three teeth; the inner tooth shorter. Maxilla blunt, with eight setae on a side, slightly sclerotized. Galea elongated, easily visible, sclerotized with three conical sensillae and one elongated sensilla at the end. Maxillar palpus is elongated, sharply narrowing toward the end, with two sensory cones in the membrane. Labium rugous and slightly sclerotized, with a wide salivary slit, and with six conspicuous setae on each side. Labial palpus short, with three conical sensillae and one elongated sensilla.

#### *Passaloecus clypealis* (Figs 5I, 5J and 7P–7R)

Mature larvae of this species were described by [20] but only very briefly, with no comments on the head and mouthparts.

Material: Czech Republic, Bohemia occ., Soseň env., Plaviště Nature Monument, reed bed at fishpond, 10 larvae; Bohemia mer., Třeboňsko Protected Landscape Area, Smržov env., loose reeds at the dam on the Smržovský Dolní rybník, 4 larvae, all from nests in reed galls installed artificially and exposed between 25.v.-30.vi.2015, P. Heneberg lgt.; Bohemia or., Železné hory Protected Landscape Area, Strádovka Nature Reserve, terrestrial reed bed from a fishpond, from nests in reed galls installed artificially and exposed between 20.v.-15.viii.2015, 4 larvae, P. Bogusch lgt., all P. Bogusch det. (coll. P. Bogusch).

Body: Length 5.32 ± 0.40 mm; width 1.00 ± 0.14 mm (n = 28). Body color pale yellowish to yellow-ochre in darker specimens. Head quite large, almost as wide as the body. The posterior parts of the segments form distinct lobes on the dorsal part, pleural lobes well developed, less developed on the prothorax and mesothorax and largest on the central abdominal segments. Bbody slightly flattened dorsoventrally. The last abdominal segment knob-like. Integument rugose with sparse but large setae. Spiracles clearly visible, small, funnel-shaped, with very a small atrium, the atrial margin thicker than the atrial pore.

Head and mouthparts: Head rounded, without any suture, width 0.76 mm, height 0.78 mm, width:height ratio ˃ 1. Head pale and unpigmented except for brownish markings on the following structures: at the end of the clypeus, labrum, mandibles, maxillae, and labium. Small antennal orbits, with three sensory cones in the membrane. Head has many pits bearing setae, mostly above the mandibular joints and on the frons. A group of eight sensillae located just above the mandibular joints. Clypeus quadratic and sclerotized at the end and two times wider than it is long; each side has two conspicuous setae and three sensillae. Labrum sclerotized with very slight emargination; each side has five large setae and seven sensillae. Mandible (length 0.29 mm) very sclerotized, with four large teeth. Maxilla sclerotized, with three big setae on each side. Galea elongated with three elongated sensory cones and one conical sensilla at the end. Maxillar palpus elongated with three elongated sensory cones at the end. Labium sclerotized, with a narrow salivary slit; each side has three large setae. Labial palpus elongated, with three elongated sensillae apically.

#### *Trypoxylon minus* (Figs 5K, 5L and 8A–8C)

Mature larvae of this species have not been described previously.

Material: Hungary bor., Pákozd env., Velencei-tó lake, terrestrial reed bed, 23.ii.2015, 3 larvae; Hungary mer., Kiskunság National Park, Munkastelep, terrestrial reed bed, 25.ii.2015, 2 larvae, all P. Bogusch et P. Heneberg lgt.; Poland bor., Lunowo, terrestrial reed bed, 30.i.2015, 3 larvae; Troszyn env., terrestrial reed bed, 30.i.2015, 3 larvae; Slowinski National Park, Rowy, terrestrial reed bed near the sea, 01.ii.2015, 4 larvae; Slowinski National Park, Gardna Wielka, terrestrial reed bed at Gardno lake, 01.ii.2015, 2 larvae; Jastarnia, terrestrial reed bed at Hel peninsula, 02.ii.2015, 2 larvae, all P. Bogusch et P. Heneberg lgt.; Czech Republic; Bohemia bor., Zahrádky, Novozámecký rybník National Natural Reserve, terrestrial reed bed surrounding a fishpond, 08.iii.2015, 17 larvae; Jestřebí env., Jestřebské slatiny National Natural Monument, terrestrial reed beds surrounding streams, 08.iii.2015, 4 larvae; Doksy env., Břehyně-Pecopala National Natural Reserve, terrestrial reed bed surrounding a fishpond, 08.iii.2015, 22 larvae; Doksy, Swamp National Natural Monument, peat bog and terrestrial reed bed, 08.iii.2015, 1 larva, all P. Bogusch et A. Astapenková lgt., all P. Bogusch det. (coll. P. Bogusch).

Body: Length 7.17 ± 0.28 mm; width 1.59 ± 0.28 mm (n = 11). Whitish, white or pale ochre in color. Body elongated, slim, and dorsoventrally flattened. Posterior parts of segments have distinct lobes; pleural lobes well developed, lobes are less developed on prothorax and most distinct and widest on last few abdominal segments. Last abdominal segment small and narrower than the head. Integument forms a fine skin with many large setae and small spinulae between them. Spiracles pale brown, atrium very faintly marked with five lines and the subatrium unarmed.

Head and mouthparts: Head width 0.89 mm, height 0.99 mm, width:height ratio < 1. Head pale and unpigmented except for brownish markings on the following structures: antennal orbits, mandibles and joints, galeae slightly pigmented, maxillar and labial palpi. Antennal orbits contain three sensory cones in the membrane. Head has many pits bearing setae, mostly on the vertex, above the clypeus, and above the mandibles; one seta also on the mandible. Clypeus only very slightly sclerotized, wide, with six conspicuous setae in one line, and six sensillae close to the base of the clypeus. Labrum slightly sclerotized, narrower than the clypeus, square-shaped, end rugous; there are nine conspicuous setae on each side. Apical margin of labrum has slight emargination in the middle. Epipharynx has many tooth-like processes laterally. Mandible (length 0.34 mm) has five small teeth and three tooth-like processes laterally. Maxilla slightly sclerotized with six conspicuous setae on each side. Galea elongated with three conical sensillae and one elongated sensilla at the end. Maxillar palpus elongated, with two elongated sensillae. Labium has a wide salivary slit, its width more than half the width of the labium, with three large setae on each side. Labial palpus elongated, with two small sensillae and one elongated sensilla at the end. Hypopharynx clearly visible and conspicuously serrated.

#### *Heriades rubicola* (Figs 6E, 6F and 8D–8F)

Mature larvae of this small species have not been previously described.

Material: Hungary bor. occ., Pákozd env., Velencei-tó lake env., terrestrial reed bed, 23.ii.2015, 3 larvae; Hungary centr., Kiskunság National Park, Izsák, Kolón-tó lake, reed bed at meadow, 24.ii.2015, 5 larvae; Kiskunság National Park, Orgovány env., terrestrial reed bed, 24.ii.2015, 20 larvae; Dunatetétlen env., Bödi-Szék, salt marsh and terrestrial reed bed, 24.ii.2015, 4 larvae; Hungary mer., Kiskunság National Park, Munkastelep env., terrestrial reed bed at saline lake, 25.ii.2015, 48 larvae; Slovenia mer., Portorož env., terrestrial reed bed near sea, 26.ii.2015, 4 larvae, P. Bogusch et P. Heneberg lgt., all P. Bogusch det. (coll. P. Bogusch).

Body: Length 5.65 ± 0.44 mm; width 1.45 ± 0.11 mm (n = 25). Color white or whitish, in some cases very slightly yellow or ochre colored. Head very small, about half of the width of the abdominal segments. Slightly dorsoventrally flattened. Posterior parts of segments form transverse welts on the dorsal part, but very poorly formed and poorly separated from other parts of the segment. Last abdominal segments rounded, narrower than other segments, and bear a transverse anus. Conspicuous, large setae on the body; more so on abdominal segments. Integument with small dense setae. Spiracles normal, atrium armed with quite a thick margin, with at least seven lines, lines not disrupted.

Head and mouthparts: Head easily visible but very small (usually smaller than half the width of the thoracic and abdominal segments) and heart-shaped, width 0.99 mm, height 0.81 mm, width:height ratio > 1. Pale and unpigmented except for brownish markings on the following structures: anterior and posterior tentorial arms, labrum, mandibles and their condyli, maxillar and labial palpi. Antennal orbits elongated along the entire segment, with two short setae at the end. Head with many pits bearing stout setae, mostly on sides and on frons above the clypeus. Slight depression in the middle of head, reaching anterior clypeal margin. Clypeus poorly visible, not well separated from other head parts, with six setae. Labrum trapezoidal and slightly sclerotized, its margin has a lobe on each side, another small lobe on each side towards the middle, as well as a small depression in the middle. Rugous structure in the middle at the base of labrum, and five setae and eight sensillae on each side of the labrum. Mandible (length 0.30 mm) sharply narrowed with three teeth, two apical teeth are blunt and the one on the outer side somewhat longer (about one width of tooth apex) than the second, the third tooth small and lateral. Mandibles have a sclerotized apex with teeth, joints and basal edge. Maxillae blunt, with many prominent setae on each side: three at the base of the galea, four around the galea, and three at the end of the maxilla. Galea elongated with two elongated sensillae at the end and one small conical sensilla. Maxillar palpus short and rudimentary. Labium with rugous structure on apical part, wide transverse salivary slit. Seven conspicuous setae on each side of labium. Labial palpus with two elongated sensillae and one small conical sensilla at the end.

#### *Stelis breviuscula* (Figs 6G and 8G–8I)

Mature larvae of this species were only briefly described by [21].

Material: Slovakia mer., Virt and Marcelová env., terrestrial reed bed at Patinský kanál, 20.i.2016, 3 larvae, P. Bogusch lgt., all P. Bogusch det. (coll. P. Bogusch).

Body: Length 5.11 ± 0.17 mm; width 1.42 ± 0.12 mm (n = 3). Color white or whitish, some parts of body (anus and head) light brown. Head very small, about half the width of the abdominal segments. Slightly dorsoventrally flattened. Posterior parts of segments form transverse welts on the dorsal side, but very poorly formed and poorly separated from other segment parts. Last abdominal segment rounded and somewhat narrower than other segments, with a transverse anus. Large conspicuous setae on body, more so on abdominal segments. Spiracles with wide margin, serrated, atrium thick-walled, with at least five septa.

Head and mouthparts: Head heart-shaped, clearly visible although very small, width 0.7 mm, height 0.61 mm, width:height ratio > 1. Pale and unpigmented except for brownish markings on the following structures: antennal orbits, labrum, mandibles with joints, maxillae with palpus and galea, labium with palpus. Very small, elongated antennal orbits with two elongated setae. Head has many pits bearing setae, mostly on sides above mandibular joints and on the vertex. Clypeus trapezoidal, slightly sclerotized, with four setae on each side of the apical part. Labrum slightly sclerotized, with lobes on each side, and a small depression in the middle. Rugous structure in the middle at the base of the labrum, seven thick setae and six sensillae on each side. Mandible (length 0.29 mm) has two teeth, one tooth shorter than the other. One prominent seta near the base of the mandible. Maxilla has tubular and elongated galea, with three sensillae at the end. Maxillar palpi elongated, narrower than the galea, with two sensillae at the end. Maxilla with at least ten big setae on each side, three of them located near the apex in front of galea. Labium small, with five setae on each side, and a wide salivary slit. Labial palpi elongated, with three sensillae at the end.

#### *Hylaeus confusus* (Figs 6A, 6B and 8J–8L)

Mature larvae of this species were previously described by [22] but the description lacked some important characteristics, e.g., the chaetotaxy.

Material: Hungary bor., Pákozd env., Velencei-tó lake env., terrestrial reed bed, 23.ii.2015, 3 larvae; Hungary occ., Fonyód env., terrestrial reed bed near Lake Balaton, 25.ii.2015, 2 larvae, all P. Bogusch et P. Heneberg lgt., all P. Bogusch det. (coll. P. Bogusch).

Body: Length 7.75 ± 0.05 mm; width 1.95 ± 0.05 mm (n = 5). Color white or whitish. Head medium large, only slightly narrower than the rest of the body. Only slightly dorsoventrally flattened. Posterior parts of segments have transverse welts on dorsal side, but they are very poorly formed and poorly separated from other segment parts. Last abdominal segment very short. Integument smooth with very few setae and sensillae. Spiracles yellowish, large, round, atrium wide, with only a thin margin, with more than seven lines.

Head and mouthparts: Head easily visible, width 1.18 mm, height 1.08 mm, width:height ratio > 1. Pale and unpigmented except for brownish markings on the following structures: antennal orbits, lateral clypeal teeth, apex of labrum, mandibles and their condyli, apical part of maxillae, galeae, labial palpi. Antennal orbits without arms and have three sensory cones in the membrane. Head has several setae, most of them on the sides above mandibular joints. Clypeus ribbed with one sclerotized tooth on each side, also three sensillae on each side. Labrum rugous, has apical margin with two very slight lobes on each side, and no emargination in the middle. Labrum with three setae on each side, with at least 16 short, stiff setae below them towards the apical part of labrum. Mandible (length 0.32 mm) with one tooth. Mandibles without joints, have sclerotized apex with tooth. Maxilla has three setae on each side, maxillar palpus large, elongated, with three sensory cones at the end. Labium with narrow salivary slit and two setae on each side. Labial palpus has two sensillae at the end. Hypopharynx rough and easily visible.

#### *Hylaeus moricei* (Figs 6C, 6D and 8M–8O)

Mature larva of this species have not been previously described, even though [22] described the nests and larvae of most of the central European species in this genus.

Material: Czech Republic, Bohemia bor. or., Zlíč env., Dubno Natural Reserve, terrestrial reed bed surroundings, 15.i.2015, 6 larvae; Bohemia bor., Zahrádky, Novozámecký rybník National Natural Reserve, terrestrial reed bed surrounding fishpond, 08.iii.2015, 3 larvae, all P. Bogusch et A. Astapenková lgt., P. Bogusch det. (coll. P. Bogusch).

Body: Length 5.46 ± 0.41 mm; width 1.26 ± 0.20 mm (n = 3). Color white or whitish. Head medium large, slightly narrower than the rest of the body. Only slightly dorsoventrally flattened. Posterior parts of segments form transverse welts on dorsal side, but very poorly formed and poorly separated from other parts of the segment. Last abdominal segment rounded. Integument smooth with only a few short setae and sensillae. Spiracles normal, atrium funnel-shaped, with a very narrow margin, with eight lines.

Head and mouthparts: Head easily visible, rounded, medium large, width 0.87 mm, height 0.79 mm, width:height ratio > 1. Pale and unpigmented except for brownish markings on the mandibles, mostly at the apex and condyli. Antennal orbits large, larger than two thirds of the length of the whole mandible, with three sensory cones in the membrane. Head almost without setae. Clypeus poorly visible, rectangular, quite narrow, with four small setae. Labrum very narrow and only slightly sclerotized, rounded without a depression in the middle. Apical part of labrum has large conspicuous setae (at least ten setae) and some sensillae. Mandible (length 0.32 mm) with one tooth that neither sharp nor blunt, and with eight small teeth on the inner side. Mandibles sclerotized only at the apex, joints sclerotized only very slightly and not darkened. Maxillae rounded at the ends with no setae, with six sensillae at each end. Galea well developed but not sclerotized, with two elongated sensillae and one small conical sensilla at the end. Maxillar palpus poorly visible and rudimentary. Labium has narrow salivary slit and wide blunt palpi, with four small setae on each side of the labium. Labial palpus has two elongated sensillae and one small conical sensilla at the end.

## Discussion

The total number of species recorded nesting in abandoned reed galls was surprisingly high and shows that these specific shelters are being used frequently by several, and occasionally by some species of aculeate Hymenoptera. These reed galls host not only nesting hymenopterans, but also insects and invertebrates of other groups, e.g., spiders, beetles, etc. Bogusch et al. [23] examined these inhabitants and showed that most of them use reed galls as a hiding or overwintering place, and some of these species are very rare across Europe. Reed galls host species bound to reed beds with long historical continuity as well as pioneer species. Among the Aculeata recorded in reed galls, several records were, in our opinion, very questionable. First was *Stenodynerus xanthomelas*, which was recorded by [12]. This species occurs in dry steppe habitats and is, in appearance, very similar to *S*. *clypeopictus*, which was first recorded as nesting in reed galls in this study. We believe that the author misidentified the species. Additionally, the records of *Pemphredon* spp. except for *P*. *fabricii* by [15] are also questionable. During four years of comprehensive studies of reed galls, we have checked more than 5,000 individuals of *Pemphredon* from reed galls and all were represented by only a single species–*P*. *fabricii*. Though the identification of species within the genus *Pemphredon* is not trivial [24–25], we assume that records of other species from this genus nesting in reed galls are based on incorrect identifications made by non-specialists. A similar situation probably exists with species of the genus *Passaloecus* other than *P*. *clypealis*. Even *P*. *clypealis* is quite a rare species, inhabiting reed galls only sporadically. Also doubtful are the records of two species of leafcutter bees (*Megachile centuncularis* and *M*. *versicolor*) published by [15]. Both of these species are very common and they use various cavities for their nesting. We have recorded nests of *M*. *versicolor* between reed stalks at an artificially made nest site (P. Bogusch, pers. obs.), but we did not find any nests of these species in reed galls examined in this study, nor in those examined previously. Finally, some species have been published under their synonyms. This situation is common especially for the genera *Pemphredon* and *Trypoxylon*, whose taxonomy has only very recently been elucidated. Records of *Trypoxylon figulus* and *T*. *attenuatum* almost certainly also represent *T*. *minus* and *T*. *deceptorium* (described by [26] and [27], respectively) and those of *Pemphredon lethifer* are represented by *P*. *fabricii* (resurrected from the synonymy by [25]).

Aculeate Hymenoptera are unique in using reed galls not only as a shelter, but also as a nesting place. Even though the galls do not have clearly visible holes in them, several species are able to access the inside through the top of the gall and make their nest inside. It is interesting that most such species use small-size prey or pollen, and nectar for their brood, and species using bigger prey usually do not use reed galls for nesting. This proposal is supported by [5], who showed that several very common reed bed species (frequently being captured in color pan traps) have never been recorded in reed galls within the reed bed sites where they occur. *Anoplius caviventris*, which catches big spiders of the genus *Clubiona*, and *Gymnomerus laevipes*, which hunts chrysomelid larvae, are probably unable to get inside the gall with these larger prey; as a result they more frequently use reed stalks, with larger openings, for nesting.

Several species nesting in reed galls are very abundant when appropriate nesting resources are available. In particular, *Pemphredon fabricii*, and also *Heriades rubicola* in southern parts of central Europe, can be found in one out of every two or every three checked galls. It remains to be determined, whether these species are specialized for nesting in reed galls or prefer reed galls over other cavities like reed stalks or plant stems. Some species, such as *Hylaeus pectoralis* or *Stenodynerus clypeopictus*, are much less common in reed galls. However, these species are also very rare in general, and form only limited populations in their habitats. These rare species are specialized to very specific habitats, such as reed beds connected with wet meadows with an abundance of flowering plants (*H*. *pectoralis*), and saline reed bed marshes (*S*. *clypeopictus*). They probably prefer reed galls for their nesting over other kinds of cavities, and *H*. *pectoralis* is usually classified in the literature as a specialist for nesting in reed galls [5–6, 12, 28] but with no evaluation or comparison to other cavity types.

The majority of the species being found in reed galls are cavity nesters with broad ecological preferences. These usually common species can be found nearly everywhere and in some cases are able to use reed galls for their nesting. Typical members of this group include *Trypoxylon minus*, *Symmorphus bifasciatus*, and *Hoplitis leucomelana*. These species are all very abundant in various habitats across Europe [3–4, 13]. The cuckoo wasp *Trichrysis cyanea* [5–6] and cuckoo bees of the genus *Stelis* (here recorded *S*. *ornatula* and *S*. *punctulatissima*) are their nest parasites. *Trichrysis cyanea* has a very broad ability to parasitize nests of many species. This species usually invades nests of wasps collecting spiders as prey for their larvae [3, 29]; however, we have occasionally found it in the nests of *Pemphredon fabricii*, provisioning its nests with aphids.

Numerous analyzed species displayed preferences for reed galls with specific parameters such as the width of the reed galls and reed stems. The widest galls were preferred by eudominant *P*. *fabricii*, but also by *Hylaeus pectoralis*, *Symmorphus bifasciatus*, and *Thyridanthrax fenestratus*. In contrast, narrower galls were more frequently occupied by *Passaloecus clypealis*, *Hylaeus moricei*, *Trypoxylon minus*, and *Stenodynerus clypeopictus* (which is not very surprising since they, especially the first two mentioned species, are very small). Importantly, the narrowest galls were not occupied by any hymenopteran species or were occupied by a negligible share of the eudominant species *P*. *fabricii*. This applied particularly to galls less than 5 mm in diameter (Fig 2A). However, species preferring large galls as well as those found in intermediate-sized galls were limited by the width of the galls as suggested by the correlation of gall width and the number of larvae present within the galls. Due to a difference in species-specific numbers of nests analyzed, this was especially prominent for the eudominant *P*. *fabricii*. However, the same relationship was also significant for less frequently examined species, such as *Trypoxylon deceptorium*, *Symmorphus bifasciatus*, and *Hoplitis leucomelana*, and even for the most common parasitoid, *Trichrysis cyanea* (Table 4). The analysis of reed stem width suggested that some species were strictly limited to thin stems. These included *Passaloecus clypealis*, *Trypoxylon minus*, and *Trypoxylon deceptorium*. Thus, these species probably represent those limited only to galls induced by *L*. *lucens* and *L*. *pulitarsis* [6, 30]. In contrast, species such as *Hylaeus moricei*, *Heriades rubicola*, *Hylaeus pectoralis*, *Symmorphus bifasciatus*, and *Thyridanthrax fenestratus* were found to be associated with larger stems, which typically occur in reed beds less stressed by drought or other factors. There were no species that preferred stems with widths ≥ 5 mm over other stem widths, despite galls on such stems being available; only *P*. *fabricii* and *Hylaeus pectoralis*, and even then only rarely, were found in galls on such stems (Fig 2B).

The length of the gall is also very important and different species settle the inner space of the gall in many ways. *Pemphredon fabricii* and *Heriades rubicola* make longest nests with higher number of brood cells than the other species, whilst both species very often extend their nest from the gall cavity into the soft innerspace among the leaves (the same was recorded several times also at *Hylaeus pectoralis*). In the contrary, *Trypoxylon minus* usually makes short nests with 1–2 brood cells even in very long and big galls. *Hylaeus pectoralis* often uses false brood cells as a defence against parasitoids. *Stenodynerus* spp. and *Trypoxylon* spp. prefer broader cavities and make short nests and thus very often occupy galls used by *Pemphredon fabricii* with some innerspace left.

Most of the species nesting in reed galls have nests very similar to one another. They use mud or sand to create bars between brood cells and also to make the closing cork for the nest. This is typical for Crabronidae and Vespidae, but atypical for bees, which use plant matter for the same purpose. Related *Hylaeus* species had nests which, in general appearance, were very similar to that recorded for the genus *Hylaeus*: only nests of small species such as *H*. *moricei* differ in size from those of larger species such as *H*. *pectoralis* and *H*. *confusus* (which are very similar and for which species identification is not trivial). The same situation applies for both species of the genus *Trypoxylon*, *T*. *deceptorium*, and *T*. *minus*, for which no species-specific differences have been found. Nest structures seem to be autapomorphic for the particular examined families since very similar nests can be found among the species of the same family or subfamily. Both species of *Stenodynerus* have nests that are also very similar to *Symmorphus bifasciatus* (all species are classified within the family Vespidae). Also, *Pemphredon fabricii* and *Passaloecus clypealis* of the subfamily Pemphredoninae (family Crabronidae) have nests very similar to each other, differing (at first sight) only in the size of brood cells and larvae. Nest structures of species described in this study correspond with previous descriptions of nests of the same species (*Trypoxylon minus* by [6]; *Passaloecus clypealis* by [7]; *Hylaeus confusus* by [22, 29]; and *Symmorphus bifasciatus* by [6]) or with descriptions of nests of phylogenetically closely related species (*Symmorphus bifasciatus* to the nest of *Symmorphus mutinensis* described by [31], *Trypoxylon minus* to the nest of *T*. *deceptorium* described by [6, 32], and *Heriades rubicola* to the nest of *H*. *truncorum* described by [21]). Nests of both species of *Stenodynerus* and *Hylaeus moricei* were described for the first time in this study.

The mature larvae show many more differences than the nests, especially with regard to the structure of mouthparts. However, comparison with previous descriptions was very problematic. Most of the larvae described in our study have not been described previously; this was true for *Chrysis angustula*, *C*. *rutilans*, *Stenodynerus chevrieranus*, *S*. *clypeopictus*, *Symmorphus bifasciatus*, *Trypoxylon minus*, *Heriades rubicola*, and *Hylaeus moricei*. Larvae of these species possess typical characteristics that are seen in the larvae of the groups [7–8, 21, 22, 31, 33–34], but are easily distinguishable from related species. For example, the larva of *Heriades rubicola* have inner mandibular teeth moved slightly laterally and possess two rows of small lateral teeth on the mandibles, which differs from those of related species of this genus by the presence of these small lateral teeth (see [21] for comparison). Larvae of previously described species correspond with the descriptions but the comparison is very problematic due to the fact that the authors of previous descriptions did not study some important characteristics, i.e., chaetotaxy (case of *Hylaeus confusus* described previously by [22] or *Stelis breviuscula* by [21]).

Mature larvae of cuckoo wasps (Chrysididae) are short and thick in general appearance, with only weak chaetotaxy on the body surface. The last few abdominal segments are the widest of the whole body. They have round heads with a wide labrum and mandibles with three sharp teeth and a typical concavity on the outer part of the mandible. *Chrysis angustula* has this concavity, but it is much less pronounced in comparison with *C*. *rutilans*; it also has markedly more sclerotized mouthparts than *C*. *rutilans*. The size of the whole body, as well as the size of various parts of the body, are very similar in both species. Also, the larvae of Vespidae, as we have described them, have three teeth on their mandibles. *Symmorphus bifasciatus* is typified by sclerotization of the margin of the labrum; its mandibles have very blunt teeth. Both species of *Stenodynerus* have a slightly bilobed labrum. They can be distinguished from each other by the shape of the labral lobes–*S*. *chevrieranus* has much narrower labrum with well-developed lobes. Its mandibular teeth are much sharper than *S*. *clypeopictus*. Spiracles of all three species of Vespidae have short and wide subatriums and a cylindrical atrium with a very narrow opening. A mature larva of *Passaloecus clypealis* is, in general appearance, very similar to larvae of *Pemphredon* and to larvae of other *Passaloecus* species described by [7]. This same author also provided a description of the larva of *P*. *clypealis*, but the description deals only with the general appearance of the body and does not comment on the mouthparts and head capsule, which are the most important characteristics. The mandible has four blunt teeth and the large maxillae and labium and clypeus are similar to those of larvae of *Pemphredon* and are typical features of larvae of *P*. *clypealis*. Larvae of other species of *Passaloecus*, as described by [7], also have four mandibular teeth but only one is apical. Only larvae of *Passaloecus corniger* and *Passaloecus eremita* have three apical teeth and one lateral, but *P*. *eremita* has very sharp, pincer-like teeth. Orientation of the teeth of larva of *P*. *corniger* is slightly different than that of the larva of *P*. *clypealis*. Larvae of *Trypoxylon* are typified by the lobes on body segments and also their mandibles have many small teeth [6, 32]. Bogusch et al. [6] found six teeth on the mandible of the larva of *T*. *deceptorium*, and five main teeth and three smaller, marginal teeth have been found on the mandible of larva of *T*. *minus*. The last segment of the body, which has the anus, has a very different shape in the two species [32]. *Heriades rubicola* has larvae characteristic of the family Megachilidae, with many setae and sensillae on the body surface, a small heart-shaped head, and mandibles with two teeth. Typical for this genus are mandibles that narrow sharply toward the apex [21], which are also present in the previously undescribed larva of *H*. *rubicola*. In contrast to other species of the genus, this species has an inner mandibular tooth placed more laterally and two rows of small tooth-like processes. Of interest is a similar difference in *Stelis breviuscula*, the only species of the genus that parasitizes the nests of *Heriades*. Species of the genus *Hylaeus* typically have nearly unsclerotized head and mandibles with only one tooth. Larvae of *H*. *confusus* typically have very blunt and robust mandibles with many small tooth-like processes on the inner side and our description corresponds with that given by [22]. The small larvae of *H*. *moricei* have sharp mandibles with only one row of very large tooth-like processes. It also differs with regard to the structure of the spiracle; the atrium is round and the opening is very narrow.

Despite the fact that reed beds are often subject to cutting, herbicide treatment, and complete eradication, the keystone species associated with such sites, frit flies *Lipara* spp., particularly *L*. *lucens*, use these sites as an important nesting resource, which allows for the survival of numerous rare species that are of interest from a conservation perspective. In this study we enlarged the list of aculeate hymenopterans associated with *Lipara*-induced reed galls to 36 nesting species, which are parasitized by another six species of aculeate parasitoids and three species of cuckoo bees. Species such as *Stenodynerus clypeopictus*, *Passaloecus clypealis*, *Rhopalum gracile*, *Hylaeus moricei*, and *Hylaeus pectoralis* can be used in nature conservation as diagnostic species relative to the quality of reed bed reservations. Prior to this study, knowledge of their nesting biology and larval morphology was almost completely absent. In this contribution, we attempted to fill in our knowledge gaps, thus providing an important tool for efficient conservation of the wetland habitats, facilitating future research, and allowing, for the first time, identification of the larvae of aculeate hymenopterans nesting in reed galls across multiple European countries.

## Supporting Information

S1 Table List of the localities with coordinates. (XLSX)
